# Methotrexate Inhibits T Cell Proliferation but Not Inflammatory Cytokine Expression to Modulate Immunity in People Living With HIV

**DOI:** 10.3389/fimmu.2022.924718

**Published:** 2022-07-29

**Authors:** Michael L. Freeman, Brian M. Clagett, Daniela Moisi, Eunice Yeh, Charles D. Morris, Angela Ryu, Benigno Rodriguez, James H. Stein, Steven G. Deeks, Judith S. Currier, Priscilla Y. Hsue, Donald D. Anthony, Leonard H. Calabrese, Heather J. Ribaudo, Michael M. Lederman

**Affiliations:** ^1^ Division of Infectious Diseases and HIV Medicine, Department of Medicine, Case Western Reserve University/University Hospitals, Cleveland Medical Center, Cleveland, OH, United States; ^2^ Center for Biostatistics in AIDS Research, Harvard T.H. Chan School of Public Health, Boston, MA, United States; ^3^ Division of Cardiovascular Medicine, Department of Medicine, University of Wisconsin School of Medicine and Public Health, Madison, WI, United States; ^4^ Division of HIV, Infectious Diseases and Global Medicine, Department of Medicine, University of San Francisco School of Medicine, San Francisco, CA, United States; ^5^ Division of Infectious Diseases, Department of Medicine, David Geffen School of Medicine, University of California, Los Angeles, Los Angeles, CA, United States; ^6^ Division of Cardiology, Department of Medicine, University of California, San Francisco School of Medicine, San Francisco, CA, United States; ^7^ Louis Stokes Cleveland Veterans Affairs Medical Center, US Department of Veterans Affairs, Cleveland, OH, United States; ^8^ Division of Rheumatic Diseases, MetroHealth Medical Center, Cleveland, OH, United States; ^9^ Fasenmyer Center for Immunology, Division of Rheumatic Diseases, Cleveland Clinic, Cleveland, OH, United States

**Keywords:** methotrexate, HIV, inflammation, T cells, proliferation, cell cycling

## Abstract

Inflammation associated with increased risk of comorbidities persists in people living with HIV (PWH) on combination antiretroviral therapy (ART). A recent placebo-controlled trial of low-dose methotrexate (MTX) in PWH found that numbers of total CD4 and CD8 T cells decreased in the low-dose MTX arm. In this report we analyzed T cell phenotypes and additional plasma inflammatory indices in samples from the trial. We found that cycling (Ki67+) T cells lacking Bcl-2 were reduced by MTX but plasma inflammatory cytokines were largely unaffected. In a series of *in vitro* experiments to further investigate the mechanisms of MTX activity, we found that MTX did not inhibit effector cytokine production but inhibited T cell proliferation downstream of mTOR activation, mitochondrial function, and cell cycle entry. This inhibitory effect was reversible with folinic acid, suggesting low-dose MTX exerts anti-inflammatory effects *in vivo* in PWH largely by blocking T cell proliferation *via* dihydrofolate reductase inhibition, yet daily administration of folic acid did not rescue this effect in trial participants. Our findings identify the main mechanism of action of this widely used anti-inflammatory medicine in PWH and may provide insight into how MTX works in the setting of other inflammatory conditions.

## Introduction

Despite suppression of human immunodeficiency virus (HIV) replication with combination antiretroviral therapy (ART), people living with HIV infection (PWH) have persistently elevated levels of immune activation and inflammation that are linked to increased risk for non-AIDS morbidities, including atherosclerotic cardiovascular disease (ASCVD) (1–3). Low-dose methotrexate (LDMTX) has been used clinically as an anti-inflammatory agent to treat a number of rheumatologic disorders and has particular utility in the treatment of rheumatoid arthritis (RA) and psoriasis, inflammatory disorders that are also associated with increased ASCVD risk (4, 5). Despite clear clinical efficacy, however, the mechanisms whereby LDMTX exerts its clinical benefit in these settings is uncertain (6). Labelled as an anti-inflammatory, its effects on inflammatory indices in plasma and immune cell phenotypes have been variable (7–11). To this end, the recently completed Cardiovascular Inflammation Reduction Trial (CIRT, NCT01594333) – a randomized, placebo-controlled clinical trial to evaluate the efficacy of LDMTX in nearly 5000 patients with stable ASCVD – found no effect of LDMTX treatment on inflammatory indices such as plasma levels of interleukin (IL)-1β, IL-6, or C-reactive protein (CRP), nor was there any treatment-mediated reduction in cardiovascular events (12). However, the CIRT trial did not include PWH, a group characterized by having elevated inflammatory indices that have been linked to morbid outcomes (3, 13). Recently, the AIDS Clinical Trials Group (ACTG) completed a randomized, double-blind, placebo-controlled trial of LDMTX in 176 ART-treated PWH to explore its anti-inflammatory and potentially cardio-protective effects (A5314, NCT01949116) (14). No important effects on plasma levels of CRP, IL-6, interferon-inducible protein (IP)-10, CD14, CD163, d-dimers, or fibrinogen were demonstrable; only plasma vascular cell adhesion molecule (VCAM) levels fell modestly in the those receiving LDMTX. Rather, the most important immunologic effects of LDMTX were significant decreases in circulating CD4 and CD8 T cell numbers (14). Immunophenotyping demonstrated that LDMTX administration was associated with significant decreases in the frequency of CD4 and CD8 T cell cycling *in vivo* as reflected by the proportions of these cells that expressed Ki-67 and also some modest decreases in T cell co-expression of CD38 and HLA-DR, markers of T cell activation (15). Although brachial artery flow-mediated dilation (FMD) was not affected by LDMTX, ultrasound imaging analysis showed that brachial artery gray level difference statistic texture-contrast (GLDS-CON) increased in the LDMTX arm and was associated with decreases in CD4 T cell numbers and D-dimer levels possibly indicating favorable arterial structure changes (16). Analysis of 2-deoxy-2-[^18^F]fluoro-D-glucose positron emission tomography with computed tomography (FDG PET/CT) imaging revealed that there was a statistically significant decrease in arterial inflammation as measured by change in the standardized uptake values for participants in the LDMTX arm (15, 17). Thus, there may be a potential benefit of LDMTX as a therapeutic agent in PWH. In this report we define the immunologic and anti-inflammatory effects and mechanisms of LDMTX administration *in vivo* and *in vitro*.

We report here the effects of LDMTX on soluble and cellular markers of inflammation and immunity in PWH enrolled in A5314 and report also the *in vitro* effects of MTX exposure on induction of immune cell inflammatory and activation indices. We found no substantial *in vivo* effects of LDMTX on soluble inflammatory indices in PWH nor could we demonstrate an effect of MTX on induction of inflammatory cytokines by T cells or monocytes *in vitro*. The reduction in T cell cycling *in vivo* after LDMTX treatment was restricted to cells that lacked the pro-survival factor Bcl-2. *In vitro* analyses revealed that MTX inhibits T cell proliferation whether induced by T cell receptor (TCR) engagement or by homeostatic cytokines in cells from PWH or HIV-uninfected controls. MTX-mediated reduction in proliferation *in vitro* was entirely reversible with folinic acid, but not by inhibition of the ecto-5’-nucleotidase CD73, which converts extracellular adenosine monophosphate (AMP) to adenosine. Thus, our data are consistent with the interpretation that LDMTX exerts its anti-inflammatory effects *in vivo* indirectly by inhibiting the proliferation and/or survival of CD4 and CD8 T cells; this appears to be due to inhibition of dihydrofolate reductase (DHFR), and not *via* release of extracellular adenosine triphosphate (ATP) or AMP as has been suggested elsewhere (6, 18, 19). Interestingly, our data suggest that daily administration of folic acid (1mg) is insufficient to rescue this anti-proliferative activity *in vivo*. Determining whether this *in vivo* effect is limited to ART-treated PWH in whom T cell cycling is robust or whether similar effects would be seen in other inflammatory settings that may not be characterized by as much T cell turnover such as RA or psoriasis will require further investigation.

## Materials and Methods

### Participant Characteristics

The overall characteristics of all participants in A5314 have been presented elsewhere (14). For this report, we performed analyses on adequately-dosed (AD) participants, who had 24-week continuous dosing with 8 or more 15mg weekly doses of low-dose methotrexate (LDMTX) or placebo. This definition includes participants who received one or more doses of 5mg LDMTX/placebo, 8 or more doses of 10mg LDMTX/placebo, and 8 or more doses of 15mg LDMTX/placebo, without any interruptions. A total of 129 participants were considered AD, 59 in the LDMTX group and 70 in the placebo group. **Table 1** summarizes the baseline characteristics for the AD participants by treatment groups. A total of 124 participants (55 LDMTX/69 placebo) had cryopreserved plasma samples available and a total of 118 participants (54 LDMTX/64 placebo) had viably cryopreserved PBMC samples. *In vitro* experiments to measure cytokine production, proliferation, and cellular activation (see below) were performed using freshly isolated or cryopreserved PBMCs from PWH on ART (n=14) or volunteers without HIV (n=24) from the Cleveland, Ohio area. As not all *in vitro* experiments were performed at the same time or with the same samples, the numbers of samples used per assay differ.

**Table 1:** Participant Characteristics.

	All	Treatment	
		LDMTX	Placebo
Total, N	129	59	70
Female, N (%)	10 (8%)	6 (10%)	4 (6%)
Median age at entry, years, (Q1, Q3)	54 (50, 60)	56 (52, 61)	53 (49, 57)
Race/Ethnicity, N (%)			
White, non-Hispanic	56 (44%)	24 (41%)	32 (46%)
Black, non-Hispanic	53 (41%)	25 (43%)	28 (40%)
Hispanic (regardless of race)	18 (14%)	9 (16%)	9 (13%)
More than one race	1 (1%)	0 (0%)	1 (1%)
Median CD4 T cell count at Entry, cells mm^-3^, (Q1, Q3)	712 (551, 917)	684 (538, 887)	729 (560, 947)
Median nadir CD4 T cells, cells mm^-3^, (Q1, Q3)	206 (98, 359)	176 (98, 356)	241 (130, 384)
Median CD8 T cell count at Entry, cells mm^-3^, (Q1, Q3)	790 (549, 1064)	809 (530, 902)	788 (582, 1136)
Entry HIV RNA < 50 copies ml^-1^, N (%)	125 (98%)	58 (98%)	69 (100%)
Type of CVD risk, N (%)			
CAD, CVD, or PAD	22 (17%)	13 (22%)	9 (13%)
Controlled Type 2 diabetes mellitus	31 (24%)	14 (24%)	17 (24%)
Smoking, hypertension, dyslipidemia, or hsCRP ≥2mg L^-1^	76 (59%)	32 (54%)	44 (63%)
10-year ASCVD risk, % (Q1, Q3)	8.8 (5.1, 13.1)	9.4 (5.1, 14.2)	8.7 (5.1, 12.9)
Current smoker, N (%)	47 (36%)	17 (29%)	30 (43%)
Medication, N (%)			
Lipid-lowering agent	82 (64%)	38 (64%)	44 (63%)
Aspirin	55 (43%)	29 (49%)	26 (37%)
Anti-hypertensive agent	90 (70%)	41 (69%)	49 (70%)
Hypoglycemic agent	30 (23%)	12 (20%)	18 (26%)

CVD, cardiovascular disease; CAD, coronary artery disease; PAD, peripheral artery disease; hsCRP, high-sensitivity C-reactive protein; ASCVD, atherosclerotic CVD.

### Flow Cytometry

As part of the original A5314 study, cryopreserved PBMC specimens from A5314 (n=118) were thawed and stained with LiveDead Yellow (ThermoFisher), CD3-BV711 (clone UCHT1, BD), CD4-AlexaFluor(AF)-700 (RPA-T4, BD), CD8-BrilliantViolet(BV)-785 (RPA-T8, BD), HLA-DR-FITC (L243, BD), CD38-APC (HIT2, BD), CD14-Pacific Blue (M5E2, BD), CD16-APC-Cy7 (3G8, BioLegend), CX3CR1-PerCP-Cy5.5 (2A9-1, BioLegend), and CD69-PE-Cy7 (L78, BD). Additional aliquots of cryopreserved PBMCs from each donor at baseline and week 24 were then thawed and stained with a second panel, consisting of LiveDead Aqua (ThermoFisher), CD3-BV605 (SK7, BD), CD4-BrilliantUltraViolet(BUV)395 (SK3, BD), CD8-APC-Cy7 (SK1, BioLegend), CD45RA-APC (HI100, BD), CD27-BV786 (O323, BioLegend), CD95-PE (DX2, BD), CD127-PECF594 (A0195D5, BioLegend), CD39-PerCPCy5.5 (eBioA1, eBioscience), CD73-BV421 (AD2, BioLegend), Ki67-PECy7 (B56, BD), and Bcl-2-FITC (Bcl-2/100, BD). All researchers remained blinded to the treatment status of the donors during flow cytometry acquisition and gating.

### Cytokine Release

Freshly isolated PBMCs from ART-treated PWH (n=6) or HIV-uninfected controls (n=6) were cultured in complete RPMI medium (RPMI (Gibco), 10% FCS (Millipore Sigma), 1% L-glutamine (Gibco), 1% Penicillin/Streptomycin (Gibco)) and stimulated with LPS (10ng ml^-1^; Invivogen), flagellin (500ng ml^-1^; Invivogen), or anti-CD3 (10μg ml^-1^; HIT3a, BD) and anti-CD28 (5μg ml^-1^; CD28.2, BD) in the presence or absence of MTX (12.5 - 100nM; Millipore Sigma). The next day, supernatants were harvested and analyzed by enzyme-linked immunosorbent assay (ELISA) for IL-1β, IL-6, IFNγ, TNF, or IL-2 (all from R&D Systems), per manufacturer’s protocol.

### *In Vitro* Assays

Cryopreserved PBMCs from PWH (n=2-6) or HIV-uninfected controls (n=2-11) were labeled with CellTraceViolet (CTV; ThermoFisher) per manufacturer’s instructions, and cultured in complete RPMI or serum-free X-VIVO 15 medium (Lonza) for 4 days while stimulated with anti-CD3 (10μg ml^-1^; HIT3a, BD) and anti-CD28 (5μg ml^-1^; CD28.2, BD), IL-2 (100U ml^-1^; Novartis), IL-7 (100ng ml^-1^; Cytheris), or IL-15 (24ng ml^-1^; R&D) in the presence or absence of MTX (12.5 - 100nM), folinic acid (0.05 – 1000μM; Millipore-Sigma), adenosine monophosphate (AMP, 1mM; Millipore Sigma), and CD73 inhibitor (α,β-methyleneadenosine 5’-diphosphate; 0.4mM, Millipore-Sigma). At the end of the culture, cells were harvested, enumerated using Liquid Counting Beads (BD), and analyzed by flow cytometry for LiveDead Aqua, CellTraceViolet, CD3-BUV737 (UCHT1), CD4-BUV395 (SK3), CD8-BV605 (SK1), CD69-PECF594 (FN50), CD25-APCH7 (M-A251) (all from BD). Assays to assess AMP/CD73 pathway activity shown in **Figures 4C, D**, were performed in X-VIVO 15 medium due to the serum sensitivity of the AMP reagent. All other assays were performed in complete RPMI.

For *in vitro* analysis of cell cycle and mitochondrial activity, cryopreserved PBMCs were labeled with CTV and cultured in complete RPMI for 4 days while stimulated with IL-15 (24ng ml^-1^; R&D) or anti-CD3 (10μg ml^-1^; HIT3a, BD) and anti-CD28 (5μg ml^-1^; CD28.2, BD), in the presence of absence of MTX (100nM) or rapamycin (250ng ml^-1^). At the end of the assay, cells were harvested and analyzed by flow cytometry. Cells were labeled with LiveDead Aqua, CD3-BUV737, CD4-BUV395, CD8-BV605, and CD25-APCH7. Then cells were fixed and permeabilized (Cytofix/Cytoperm, BD) and stained with antibodies to Ki67-PECy7 (B56, BD), TFAM-AF488 (18G102B2E11, Abcam), and COX IV-AF568 (EPR9442(ABC), Abcam).

For detection of phosphorylated ribosomal S6 protein and STAT5, cryopreserved PBMCs from HIV-uninfected controls (n=3) were cultured in complete RPMI medium for 45 minutes at 37°CC, stimulated with IL-15 (24ng ml^-1^) or anti-CD3/anti-CD28 in the presence or absence of MTX (100nM) or rapamycin (250ng ml^-1^). After the culture, cells were washed and fixed with 16% formaldehyde for 10 minutes at 37°C, washed and treated with 90% methanol for 20 minutes at -20°C, then washed and incubated for 20 minutes with anti-S6 pS240-APC (REA420, Miltenyi Biotec) and anti-STAT5 pY694-PE (47/Stat5[pY694], BD).

For the measurements of MTX uptake, cryopreserved PBMCs from HIV-uninfected controls (n=5) were cultured with anti-CD3/anti-CD28 or medium overnight in complete RPMI at 37°C. Folinic acid (1μM) and/or fluorescein-labeled MTX (100nM, ThermoFisher) were added and after 1h, cells were harvested, washed, and analyzed by flow cytometry. Cells were labeled with LiveDead Aqua, CD3-BUV737, CD4-BUV395, and CD8-BV605. The fluorescein-MTX signal was calculated by subtracting the mean fluorescence intensity (MFI) of unlabeled cells from the MFI of cells treated with fluorescein-MTX.

### Statistics

Given their skewed distribution, all plasma biomarker outcomes were analyzed on the log10 scale. For interpretation, percentile statistics (median, 1st quartile, 3rd quartile) of measured values are presented on the untransformed scale. Cellular markers were analyzed on the original measured scale. Specifically, the parameter of interest is the percentage of the parent cells that expressed the given marker. All treatment group comparisons use a Wilcoxon rank sum test. All p-values are presented at the nominal level with no formal adjustment for multiple comparisons. Given the number of comparisons being performed and the exploratory nature of these analyses, marginal p-values should be interpreted cautiously, with attention to the consistency of the data distribution over time and to comparable outcome measures, as well as biological plausibility. The alpha level of 0.05 is used as an informal screening for significance, and the text does not necessarily highlight all p-values below this level. All statistics reported in **Tables 1**–**3**, and **Supplementary Tables 1, 2** were performed using SAS software (version 9.4).

For *in vitro* studies, comparisons between two groups used nonparametric Mann-Whitney U tests. Comparisons among three or more groups were performed with nonparametric Kruskal-Wallis tests with Dunn’s multiple comparison or nonparametric Friedman’s test for multiple paired comparisons. For MTX titration experiments, whether one curve could fit both groups, and the MTX 50% inhibitory concentrations (IC50s) for each group were determined using variable slope nonlinear regression, log(inhibitor) vs. normalized response. Determination of whether slopes were significantly nonzero was performed by simple linear regression. All statistics for *in vitro* studies were performed using Prism software (version 9.2, GraphPad).

### Study Approval

All participants in the ACTG A5314 clinical trial (NCT01949116) were enrolled after written informed consent was obtained at each clinical site in accordance with the Declaration of Helsinki. All *in vitro* experiments were approved by the Institutional Review Board (IRB) of University Hospitals, Cleveland Medical Center (IRB #01-98-55). Blood samples were acquired with written informed consent in accordance with the Declaration of Helsinki.

## Results

### Effects of LDMTX on Inflammation, Immune Cell Activation, and T Cell Cycling *In Vivo*

In A5314, 24 weeks of LDMTX treatment had no effects on plasma levels of CRP, IL-6, IP-10, CD14, CD163, d-dimers, or fibrinogen; and VCAM levels fell only modestly (14). Among participants who had 24-week continuous dosing with 8 or more 15mg doses of LDMTX or placebo (adequately-dosed; **Table 1**), we found no differences in the proportions of monocyte subsets nor differences in their expression of CD69 or fractalkine receptor (**Supplementary Figures 1A–C**, **Supplementary Table 1**), suggesting LDMTX had no effect on circulating monocytes, a key proinflammatory component of the innate cellular immune response. We next measured the plasma levels of two more biomarkers linked to inflammatory states at week 0 (baseline) and week 24: fractalkine (CX3CL1), a marker of endothelial dysfunction, and IL-18, a marker of inflammasome activation (**Supplementary Table 2**), and found no differences of these markers between placebo and LDMTX arms.

LDMTX treatment in A5314 did however decrease the absolute numbers of circulating CD4 and CD8 T cells (14), and reduced the proportion of T cells in cell cycle (15). Here we further explored the *in vivo* effects of LDMTX by measuring the expression of cellular indices of activation, including CD38 and HLA-DR coexpression, CD39, CD73, CD95, CD127, PD-1, and CX3CR1 at baseline and week 24 among adequately-dosed A5314 participants (**Supplementary Figures 1A, D–F**, **Table 2**). We found modest but consistent and statistically significant reductions in CD38 and HLA-DR coexpression, and CD39 expression, on total CD4 and CD8 T cells in the LDMTX arm by week 24. We also observed slight increases in expression of the ecto-5’-nucleotidase CD73 on both CD4 and CD8 T cells in the LDMTX group. Plasma levels of β2 microglobulin, a soluble marker of T cell activation, were not different between placebo and LDMTX arms (**Supplementary Table 2**).

**Table 2:** Changes from baseline in T cell activation markers.

	Treatment				P-value Week 24
	LDMTX (N=54)	Placebo (N=64)	LDMTX (N=54)	Placebo (N=64)	
	Week 0	Week 0	Δ to Week 24	Δ to Week 24	
CD4 T cells					
CD38+HLA-DR+, % (Q1, Q3)	2.20 (1.40, 3.30)	1.90 (1.50, 2.70)	-0.20 (-0.85, 0.10)	0.00 (-0.30, 0.50)	**0.007**
CD39+, % (Q1, Q3)	6.90 (4.20, 9.50)	6.90 (3.90, 9.50)	-1.15 (-2.60, -0.10)	0.00 (-0.60, 0.35)	**<0.001**
CD73+, % (Q1, Q3)	14.4 (10.3, 19.4)	12.4 (9.70, 18.7)	0.70 (0.10, 1.60)	0.00 (-0.65, 0.45)	**<0.001**
CD95+, % (Q1, Q3)	62.0 (51.3, 74.5)	64.1 (50.5, 76.4)	-1.70 (-4.40, 0.40)	-1.00 (-4.00, 1.35)	0.45
CD127+, % (Q1, Q3)	88.7 (83.2, 91.2)	88.8 (84.7, 91.2)	1.25 (0.00, 2.40)	0.60 (-0.75, 1.80)	**0.025**
PD-1+, % (Q1, Q3)	39.9 (33.1, 49.2)	42.8 (32.8, 51.6)	-2.30 (-4.00, 0.70)	-1.05 (-3.40, 1.20)	0.14
CX3CR1+, % (Q1, Q3)	7.30 (5.60, 10.5)	7.50 (6.00, 11.3)	-0.30 (-1.15, 0.60)	-0.20 (-1.30, 0.65)	0.92
CD8 T cells					
CD38+HLA-DR+, % (Q1, Q3)	2.90 (1.60, 5.10)	3.10 (1.90, 5.70)	-1.30 (-2.40, -0.40)	-0.10 (-0.60, 1.00)	**<0.001**
CD39+, % (Q1, Q3)	1.80 (0.90, 3.40)	1.70 (0.95, 2.80)	-0.50 (-1.00, -0.10)	-0.05 (-0.30, 0.20)	**<0.001**
CD73+, % (Q1, Q3)	36.0 (28.7, 48.2)	39.3 (28.3, 49.0)	2.80 (0.70, 5.40)	0.90 (-2.70, 3.05)	**0.001**
CD95+, % (Q1, Q3)	84.7 (76.7, 88.0)	81.5 (75.0, 89.1)	-1.50 (-3.20, 0.10)	-0.45 (-2.75, 0.90)	0.13
CD127+, % (Q1, Q3)	63.7 (50.5, 74.7)	63.0 (51.8, 72.2)	1.55 (-0.80, 5.00)	1.25 (-4.15, 4.05)	0.07
PD-1+, % (Q1, Q3)	36.1 (26.6, 47.5)	38.4 (27.8, 47.2)	-1.65 (-3.50, 0.30)	-0.90 (-3.30, 1.15)	0.54
CX3CR1+, % (Q1, Q3)	24.3 (18.2, 35.4)	27.1 (17.1, 34.3)	-1.05 (-3.35, 2.25)	0.15 (-3.90, 4.50)	0.36

Bold values show P-values that are statistically significant (<0.05).

Plasma levels of the homeostatic cytokine IL-7 are typically elevated during lymphopenia and may contribute to cell cycling and proliferation. Plasma IL-7 levels were unaffected by LDMTX (**Table S2**), suggesting that the effects of LDMTX on cell numbers and cycling were not due to alterations in this homeostatic cytokine but may be intrinsic to the T cells. We measured the proportions of total CD4 and CD8 T cell maturation subsets and the proportions of these subsets in cell cycle (Ki67+) and expressing the anti-apoptosis molecule Bcl-2 at baseline, and the change from baseline to week 24 of treatment (**Supplementary Figure 1G**, **Table 3**). We found no apparent differences in the maturation subset distribution among CD4 T cells. For CD8 T cells, we found a significant reduction in the proportion of effector memory (TEM) cells, which are the most enriched for cycling Ki67+ cells. In general, we observed reductions in the proportions of Ki67+ cells and increases in proportion of Bcl-2+ cells, suggesting that the cells that remain after LDMTX treatment are enriched for cells with mechanisms to preserve viability. Accordingly, in all T cell subsets we examined, the reductions in Ki67+ cells were entirely within the Bcl-2- population, and not of Ki67+ cells that co-expressed Bcl-2 (**Table 3**). Taken together, our data suggest that the reduction in activated and cycling T cells *in vivo* might be due to impaired survival of cells activated to enter cell cycle.

**Table 3:** Changes from baseline in T cell subsets and Ki67 and Bcl-2 expression.

	Treatment				P-value Week 24
	LDMTX (N=54)	Placebo (N=64)	LDMTX (N=54)	Placebo (N=64)	
	Week 0	Week 0	Δ to Week 24	Δ to Week 24	
CD4 T cells					
Ki67+	3.10 (2.30, 4.10)	3.00 (2.50, 3.65)	-0.55 (-1.10, 0.10)	0.10 (-0.45, -0.65)	**<0.001**
Bcl-2+	92.8 (90.3, 95.0)	92.3 (89.9, 94.4)	1.40 (0.30, 2.30)	-0.35 (-1.10, 0.60)	**<0.001**
Ki67+Bcl-2+	1.45 (1.00, 1.90)	1.25 (1.00, 1.60)	-0.05 (-0.40, 0.20)	0.00 (-0.20, 0.25)	0.47
Ki67+Bcl-2-	1.70 (1.30, 2.10)	1.75 (1.30, 2.25)	-0.50 (-0.80, -0.10)	0.10 (-0.20, 0.45)	**<0.001**
Naïve (CD45RA+CD27+)	41.5 (32.1, 53.2)	39.9 (28.0, 54.3)	1.30 (-0.80, 3.70)	1.00 (-1.05, 3.65)	0.62
…. Ki67+	0.56 (0.35, 0.69)	0.50 (0.36, 0.64)	-0.03 (-0.12, 0.06)	0.02 (0.10, 0.15)	0.20
…. Bcl-2+	97.2 (95.6, 98.2)	96.4 (94.2, 98.1)	0.31 (-0.31, 0.62)	-0.16 (-1.11, 0.34)	**<0.001**
…. Ki67+Bcl-2+	0.40 (0.26, 0.57)	0.32 (0.26, 0.47)	0.01 (-0.08, 0.09)	0.01 (-0.07, 0.08)	0.87
…. Ki67+Bcl-2-	0.12 (0.08, 0.18)	0.13 (0.09, 0.19)	-0.03 (-0.08, 0.01)	0.01 (-0.02, 0.05)	**0.003**
TEMRA (CD45RA+CD27-)	1.10 (0.30, 2.80)	1.55 (0.60, 4.65)	0.05 (-0.20, 0.20)	0.00 (-0.40, 0.40)	0.41
…. Ki67+	3.40 (2.38, 5.31)	2.86 (1.89, 4.84)	-0.59 (-1.42, 0.23)	0.56 (-0.49, 1.80)	**<0.001**
…. Bcl-2+	94.0 (88.2, 96.1)	93.2 (88.3, 95.7)	2.71 (1.40, 4.78)	-0.45 (-2.59, 1.68)	**<0.001**
…. Ki67+Bcl-2+	1.88 (1.38, 2.73)	1.54 (1.04, 2.43)	0.25 (-0.55, 0.84)	0.36 (-0.32, 0.97)	0.47
…. Ki67+Bcl-2-	1.36 (0.63, 2.95)	1.17 (0.71, 2.16)	-0.69 (-1.34, -0.30)	0.17 (-0.28, 0.68)	**<0.001**
TCM (CD45RA-CD27+)	45.0 (39.1, 55.3)	46.0 (36.4, 56.3)	-0.35 (-3.60, 1.50)	-0.55 (-3.95, 0.90)	0.91
…. Ki67+	4.83 (3.91, 6.13)	4.59 (4.02, 5.78)	-0.68 (-1.63, 0.15)	0.21 (-0.84, 1.26)	**0.002**
…. Bcl-2+	90.2 (88.1, 91.9)	89.2 (86.2, 91.4)	2.03 (0.46, 3.61)	-0.46 (-1.83, 0.70)	**<0.001**
…. Ki67+Bcl-2+	2.08 (1.47, 2.82)	1.81 (1.45, 2.44)	-0.14 (-0.61, 0.24)	0.03 (-0.38, 0.39)	0.23
…. Ki67+Bcl-2-	2.95 (2.28, 3.66)	2.79 (2.47, 3.39)	-0.63 (-1.28, 0.11)	0.09 (-0.41, 0.73)	**<0.001**
TEM (CD45RA-CD27-)	6.85 (4.80, 13.8)	8.80 (4.95, 12.1)	-0.60 (-1.30, 0.20)	-0.40 (-1.25, 0.40)	0.67
…. Ki67+	6.59 (5.09, 10.3)	6.47 (4.64, 8.10)	-1.40 (-2.93, 0.04)	0.55 (-1.21, 2.19)	**<0.001**
…. Bcl-2+	89.4 (84.8, 91.3)	88.1 (83.6, 91.4)	4.54 (1.61, 6.73)	-0.10 (-3.41, 1.82)	**<0.001**
…. Ki67+Bcl-2+	3.00 (2.11, 3.91)	2.42 (1.74, 3.31)	0.09 (-0.65, 0.62)	0.16 (-0.55, 0.59)	0.78
…. Ki67+Bcl-2-	3.76 (2.85, 6.16)	3.76 (2.56, 4.79)	-1.73 (-2.89, -0.55)	0.31 (-0.66, 1.45)	**<0.001**
CD8 T cells					
Ki67+	2.30 (1.70, 2.70)	1.95 (1.55, 2.50)	-0.50 (-1.00, -0.20)	0.10 (-0.30, 0.50)	**<0.001**
Bcl-2+	91.9 (89.2, 93.7)	89.8 (86.4, 93.7)	2.75 (1.70, 4.70)	-0.15 (-2.00, 1.55)	**<0.001**
Ki67+Bcl-2+	1.10 (0.80, 1.50)	1.00 (0.70, 1.30)	0.00 (-0.30, 0.20)	0.00 (-0.15, 0.30)	0.49
Ki67+Bcl-2-	1.00 (0.70, 1.30)	0.80 (0.65, 1.30)	-0.60 (-0.90, -0.30)	0.10 (-0.20, 0.20)	**<0.001**
Naïve (CD45RA+CD27+)	19.5 (14.6, 27.9)	21.6 (16.8, 28.7)	0.75 (-0.20, 2.80)	0.30 (-1.40, 2.90)	0.31
…. Ki67+	0.42 (0.28, 0.57)	0.41 (0.27, 0.60)	-0.06 (-0.12, 0.08)	0.00 (-0.07, 0.14)	**0.029**
…. Bcl-2+	96.1 (94.0, 97.3)	95.0 (91.3, 97.2)	0.86 (-0.18, 1.91)	0.22 (-0.81, 1.11)	**0.027**
…. Ki67+Bcl-2+	0.29 (0.18, 0.40)	0.28 (0.19, 0.41)	0.01 (-0.05, 0.10)	0.01 (-0.04, 0.07)	0.92
…. Ki67+Bcl-2-	0.10 (0.07, 0.18)	0.11 (0.07, 0.18)	-0.05 (-0.09, -0.02)	0.00 (-0.05, 0.06)	**<0.001**
TEMRA (CD45RA+CD27-)	25.6 (17.3, 36.8)	26.1 (15.1, 36.3)	0.15 (-3.30, 2.10)	-1.00 (-3.75, 2.65)	0.77
…. Ki67+	2.07 (1.51, 2.82)	1.95 (1.57, 2.32)	-0.13 (-0.65, 0.47)	0.15 (-0.39, 0.65)	0.12
…. Bcl-2+	92.6 (89.8, 95.0)	91.5 (86.9, 94.1)	2.19 (1.11, 3.87)	-0.25 (-2.78, 1.81)	**<0.001**
…. Ki67+Bcl-2+	1.41 (1.07, 2.04)	1.24 (0.93, 1.60)	0.17 (-0.26, 0.66)	0.14 (-0.34, 0.42)	0.28
…. Ki67+Bcl-2-	0.59 (0.43, 0.84)	0.55 (0.37, 0.91)	-0.30 (-0.48, -0.10)	0.05 (-0.13, 0.24)	**<0.001**
TCM (CD45RA-CD27+)	34.6 (27.2, 42.9)	33.3 (21.8, 42.6)	-0.60 (-2.70, 2.00)	-0.45 (-4.95, 2.50)	0.55
…. Ki67+	2.25 (1.73, 3.24)	2.23 (1.52, 3.05)	-0.76 (-1.31, -0.16)	0.13 (-0.18, 0.62)	**<0.001**
…. Bcl-2+	90.0 (86.0, 92.7)	87.3 (83.5, 81.2)	3.34 (1.82, 5.42)	0.34 (-2.04, 1.77)	**<0.001**
…. Ki67+Bcl-2+	0.94 (0.64, 1.40)	0.84 (0.61, 1.18)	-0.08 (-0.28, 0.14)	0.03 (-0.17, 0.22)	0.09
…. Ki67+Bcl-2-	1.28 (0.86, 1.86)	1.20 (0.85, 1.89)	-0.71 (-1.08, -0.29)	0.10 (-0.13, 0.43)	**<0.001**
TEM (CD45RA-CD27-)	12.4 (9.00, 18.2)	13.2 (8.75, 18.9)	-0.90 (-1.80, 1.20)	0.50 (-0.90, 1.85)	**0.018**
…. Ki67+	4.16 (3.36, 6.54)	4.20 (2.96, 5.40)	-1.35 (-2.69, -0.57)	-0.01 (-0.77, 1.08)	**<0.001**
…. Bcl-2+	88.7 (84.1, 90.7)	87.0 (82.0, 90.7)	4.34 (2.85, 7.49)	0.17 (-2.09, 2.27)	**<0.001**
…. Ki67+Bcl-2+	2.05 (1.66, 2.94)	1.62 (1.26, 2.72)	-0.03 (-0.52, 0.45)	0.03 (-0.52, 0.61)	0.51
…. Ki67+Bcl-2-	2.22 (1.57, 3.38)	2.17 (1.44, 3.21)	-1.35 (-2.26, -0.82)	0.06 (-0.52, 0.55)	**<0.001**

TCM, central memory T cells; TEM, effector memory T cells; TEMRA, effector memory T cells that have reacquired CD45RA expression.

Bold values show P-values that are statistically significant (<0.05).

### Effects of MTX on Inflammation and T Cell Activation *In Vitro*

**Figure 1:**
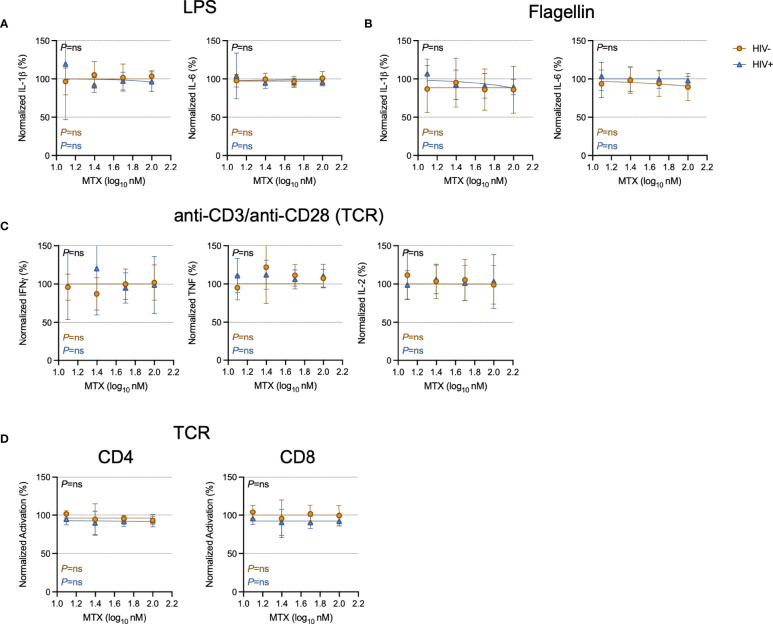
Minimal effect of MTX on cytokine production and activation *in vitro*. Freshly isolated PBMCs from PWH (HIV+, blue triangles, n=6) or HIV-uninfected controls (HIV-, gold circles, n=6) were stimulated overnight with LPS (10ng ml^-1^) **(A)**, flagellin (500ng ml^-1^) **(B)**, or anti-CD3 (10μg ml^-1^) and anti-CD28 (5μg ml^-1^)(TCR)**(C)** in the presence or absence of MTX at indicated concentrations. At the end of the culture, supernatants were collected and analyzed by ELISA for indicated cytokines. **(D)** Cryopreserved PBMCs from PWH (blue triangles, n=6) or HIV-uninfected controls (gold circles, n=8-11) were stimulated for 4 days with anti-CD3 (10μg ml^-1^) and anti-CD28 (5μg ml^-1^)(TCR) in the presence or absence of MTX at indicated concentrations. At the end of the culture, CD4 (left) and CD8 (right) T cells were analyzed by flow cytometry for expression of activation markers CD69 and CD25. Data were normalized to medium-only control values. P-values in black indicate whether one curve fits both HIV- and HIV+ donors; variable slope non-linear regression, log(inhibitor) vs. normalized response. P-values in color indicate whether the slope of each curve is significantly non-zero; simple linear regression. ns, not significant.

To model the effects of how the clinical anti-inflammatory effect of LDMTX might be mediated, we stimulated freshly isolated peripheral blood mononuclear cells (PBMCs) from healthy controls or from PWH for 24 hours with lipopolysaccharide (LPS) and flagellin to activate cells *via* toll-like receptors (TLR) 4 and TLR5, respectively, and measured levels of IL-1β and IL-6 in the culture supernatant by enzyme-linked immunosorbent assay (ELISA, **Figures 1A, B**). We found no effect of MTX on IL-1β or IL-6 production over a range of concentrations, from 12.5nM to 100nM.

To determine if MTX inhibited T cell activation *in vitro*, we stimulated freshly isolated PBMCs from PWH or from control donors with anti-CD3 and anti-CD28 to polyclonally activate T cells *via* the T cell receptor (TCR) for 24 hours, then measured culture supernatant levels of interferon-gamma (IFNγ), tumor necrosis factor (TNF), and IL-2 by ELISA. We found no effect of MTX over a range of concentrations, from 12.5nM to 100nM (**Figure 1C**). We then stimulated cryopreserved PBMCs with anti-CD3/anti-CD28 for 4 days in the presence or absence of MTX and measured expression of the activation markers CD69 and CD25 on CD4 and CD8 T cells (**Figure 1D**). MTX exposure had no significant effect on T cell activation after stimulation through the TCR.

### MTX Inhibits Proliferation of Activated T Cells

**Figure 2:**
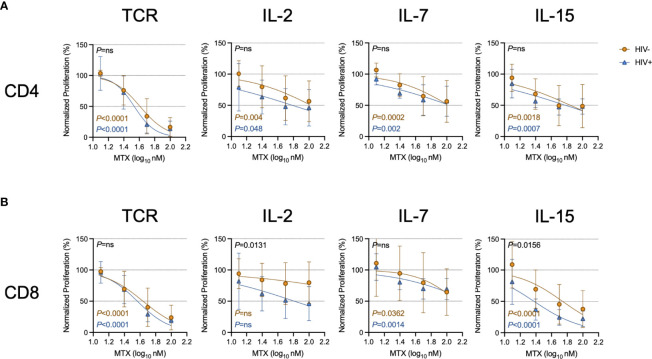
MTX inhibits T cell proliferation *in vitro*. Cryopreserved PBMCs from PWH (blue triangles, n=6) or HIV-uninfected controls (gold circles, n=8-11) were labeled with CellTraceViolet (CTV) then stimulated for 4 days with anti-CD3 (10μg ml^-1^) and anti-CD28 (5μg ml^-1^)(TCR), IL-2 (100U ml^-1^), IL-7 (100ng ml^-1^), or IL-15 (24ng ml^-1^) in the presence or absence of MTX at indicated concentrations. At the end of the culture, CD4 **(A)** and CD8 **(B)** T cells were analyzed by flow cytometry for CTV dilution. Data were normalized to medium-only control values. P-values in black indicate whether one curve fits both HIV- and HIV+ donors; variable slope non-linear regression, log(inhibitor) vs. normalized response. P-values in color indicate whether the slope of each curve is significantly non-zero; simple linear regression. ns, not significant.

Given the effects of LDMTX on T cell numbers and cycling *in vivo* (14, 15) (**Table 3**), we next measured the effect of MTX on proliferation induced by TCR engagement or by the cytokines IL-2, IL-7, and IL-15. The proportions of CellTraceViolet (CTV)-labeled CD4 and CD8 T cells that proliferated were significantly reduced in the presence of MTX in nearly all conditions after 4 days of culture (**Figure 2**). The MTX-mediated reduction in CD8 T cell proliferation following IL-2 exposure did not reach significance for cells from either PWH or controls, and in that condition, CD8 T cells from PWH seemed to be slightly more sensitive to the effects of MTX, as they were following IL-15 exposure (**Supplementary Table 3**). Taken together, our results show that MTX has minimal effect on T cell activation but substantially inhibits the proliferation of activated cells *in vitro* and *in vivo*, particularly for cells strongly stimulated through the TCR.

### MTX Inhibits T Cell Proliferation After Cell Cycle Entry and Folinic Acid, but Not CD73 Activity Blockade, Reverses MTX-Induced Inhibition

**Figure 3:**
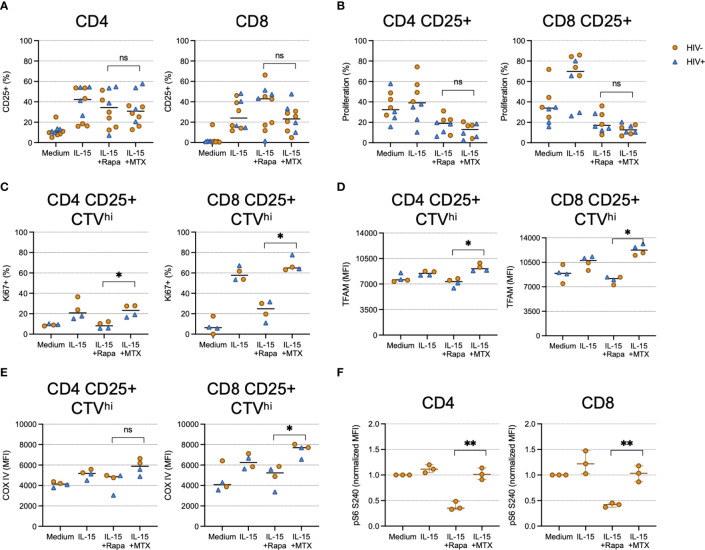
MTX inhibits IL-15-induced proliferation at a step after cell cycle entry and mTOR activity. Cryopreserved PBMCs from PWH (blue triangles, n=2-4) or HIV-uninfected controls (gold circles, n=2-6) were labeled with CellTraceViolet (CTV) then stimulated for 4 days **(A–E)** or 45 minutes **(F)** with IL-15 (24ng ml^-1^) in the presence or absence of MTX (100nM) or rapamycin (rapa; 250ng ml^-1^). At the end of the culture, cells were harvested and analyzed by flow cytometry. **(A)** The proportion of CD4 (left) and CD8 (right) T cells expressing CD25. **(B)** The proportion of CD4 (left) and CD8 (right) CD25+ cells that diluted CTV dye. **(C)** The proportion of CD4 (left) and CD8 (right) cells that did not dilute CTV dye (CTV^hi^) that expressed Ki67. **(D)** The mean fluorescence intensity (MFI) of mitochondrial TFAM among CD4 (left) and CD8 (right) CD25+ CTV^hi^ cells. **(E)** The MFI of mitochondrial COX IV among CD4 (left) and CD8 (right) CD25+ CTV^hi^ cells. **(F)** Normalized MFI of ribosomal S6 protein phosphorylated at S240 among CD4 (left) and CD8 (right) T cells. Statistical significance determined between IL-15+rapa and IL-15+MTX groups; Mann-Whitney test. ns, not significant; *P < 0.05; **P < 0.01.

We next wanted to investigate the mechanisms of MTX-mediated proliferation inhibition. We and others have shown that CD8 T cell proliferation induced by IL-15 requires mechanistic target of rapamycin (mTOR) activity and can be inhibited by rapamycin (20, 21). To determine if the anti-proliferative effects of MTX exposure were mediated *via* mTOR pathway inhibition, we stimulated CTV-labeled CD4 and CD8 T cells with IL-15 for 4 days in the presence or absence of MTX or rapamycin. Neither compound affected IL-15-induced STAT5 phosphorylation (**Supplementary Figure 2**) or cellular activation, as measured by CD25 expression (**Figure 3A**), but both rapamycin and MTX inhibited the proliferation of CD25+ cells, as measured by CTV dye dilution (**Figure 3B**). LDMTX treatment *in vivo* was associated with a loss of Ki67+ T cells, but whether activated T cells were inhibited from entry into the cell cycle or were lost following attempted cell division could not be determined from the *in vivo* data. Therefore, we next measured intracellular Ki67 expression in activated cells that had not diluted the CTV dye (CTV^hi^, **Figure 3C**). While rapamycin inhibited cell cycle entry, cells exposed to MTX had abundant Ki67 expression, indicating intact entry into the cell cycle. Similarly, levels of the mitochondrial proteins transcriptional factor A, mitochondrial (TFAM)(**Figure 3D**) and cytochrome c oxidase subunit 4 (COX IV)(**Figure 3E**) were intact in IL-15-stimulated cells cultured with MTX but not rapamycin. Thus, while mTOR pathway inhibition by rapamycin disrupts mitochondrial function and cell cycle entry following IL-15 exposure, MTX blocks IL-15-induced proliferation without impairing mitochondrial activity or cell cycle entry. This conclusion was confirmed by measuring phosphorylation of ribosomal protein S6, an event downstream of IL-15-stimulated mTOR activity; MTX did not impair S6 phosphorylation, while, as expected, rapamycin did (**Figure 3F**). Similarly, we observed elevated cycling and mitochondrial function within activated yet nonproliferating cells exposed to MTX when stimulated through TCR engagement, whereas rapamycin notably inhibited TCR-induced mitochondrial activity, but only minimally affected proliferation after 4 days of culture (**Supplementary Figure 3**).

**Figure 4:**
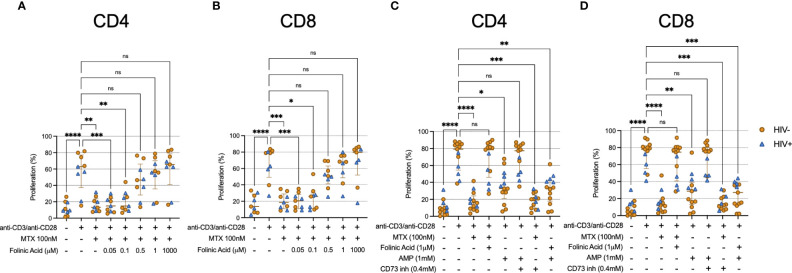
Folinic acid restores proliferation inhibited by MTX. **(A, B)** Cryopreserved PBMCs from PWH (blue triangles, n=4) or HIV-uninfected donors (gold circles, n=5) were labeled with CellTraceViolet (CTV) then stimulated for 4 days with anti-CD3/anti-CD28 in the presence or absence of MTX (100nM) and folinic acid (0.05 – 1000μM). At the end of the culture, CD4 **(A)** and CD8 **(B)** T cells were analyzed by flow cytometry for CTV dilution. **(C, D)** Cryopreserved PBMCs from PWH (blue triangles, n=4) or HIV-uninfected donors (gold circles, n=9) were labeled with CellTraceViolet then stimulated for 4 days with anti-CD3/anti-CD28 (TCR) in the presence or absence of MTX (100nM), folinic acid (1mM), adenosine monophosphate (AMP, 1mM), or CD73 inhibitor (0.4mM). At the end of the culture, CD4 **(C)** and CD8 **(D)** T cells were analyzed by flow cytometry for CTV dilution. Statistical significance determined among all groups using nonparametric paired Friedman’s test. ns, not significant; *P < 0.05; **P < 0.01; ***P < 0.001; ****P < 0.0001.

It is well appreciated that MTX inhibits the activity of DHFR. To determine whether this pathway was relevant to the effects of MTX in our assays, we cultured TCR-activated CD4 and CD8 T cells with MTX *in vitro* in the presence of various concentrations of folinic acid. TCR-induced proliferation was substantially restored in the presence of 0.5μM folinic acid (**Figures 4A, B**). Importantly, folinic acid and MTX utilize the same cellular receptor, reduced folate carrier 1 (RCF1) (18). Thus, experiments using MTX and folinic acid could be open to misinterpretation if the effect of folinic acid was due to its competition with MTX for RCF1. To address this concern, we performed an *in vitro* MTX uptake assay. We found similar uptake of fluorescein-labeled MTX by TCR-stimulated T cells in the absence or presence of 1μM folinic acid (**Supplementary Figure 4**) – a concentration that fully inhibited the anti-proliferative effect of MTX in our other assays – consistent with the interpretation that the *in vitro* restoration of TCR-induced proliferation by folinic acid was not due to interference with MTX uptake. It has been postulated that MTX may inhibit cellular function *via* the extracellular accumulation of adenosine, and our data showing increased T cell expression of CD73 following LDMTX administration in the A5314 participants (**Table 2**) suggested that this pathway might be important for the reduced T cell numbers *in vivo*. CD73 converts extracellular AMP to adenosine, which can then enter the cell *via* adenosine receptors and inhibit cellular processes, and when we exposed TCR-stimulated T cells to AMP, proliferation was inhibited in a CD73-dependent manner (**Figures 4C, D**). However, blocking CD73 could not restore proliferation inhibited by MTX, nor could folinic acid restore AMP-mediated inhibition. Thus, our data are consistent with the interpretation that MTX impairs activation-induced T cell proliferation *via* inhibition of DHFR and not by the accumulation of extracellular adenosine. This inhibitory activity is downstream of cellular activation, mTOR activation, mitochondrial function, and cell cycle entry.

## Discussion

Administration of MTX in high doses has been used to treat malignancies, and in low-doses, MTX is widely used as an anti-inflammatory agent in rheumatologic diseases, although how exactly LDMTX confers its clinical efficacy has been unclear (6). Notably, LDMTX had no apparent anti-inflammatory effect in either the CIRT trial of nearly 5000 participants with stable ASCVD or in our own trial of 129 adequately-dosed PWH on ART with sustained virus suppression – in both studies, plasma levels of IL-1β, IL-6, and CRP all remained stable (12, 14). Intriguingly, PWH who received LDMTX had significant decreases in the numbers of circulating CD4 T cells at week 24 only (-58 CD4 T cells μl^-1^ for LDMTX versus +2 for placebo, P=0.016) and more significant decreases in CD8 T cells at weeks 8, 12, and 24 (-103 CD8 T cells μl^-1^ for LDMTX versus -2 for placebo, P=0.001 at week 24).

In this follow-up study to A5314, we further characterized the effects of LDMTX on indices of inflammation and immunity in PWH, finding a substantial reduction in expression of the cell cycle marker Ki67 among the T cells that remained after LDMTX. Importantly, loss of Ki67 was restricted to cells lacking the anti-apoptotic molecule Bcl-2. In other words, cell cycling capacity was seemingly retained among cells protected from undergoing apoptosis. Alternatively, Ki67+ cells in cycle that expressed Bcl-2 might have been inhibited from progressing to the S phase and therefore not be as susceptible to the DHFR-inhibiting effects of MTX (22, 23). We propose that cellular progression to cell division after cell cycle entry in the absence of Bcl-2 and in the presence of MTX (and thus, the absence of DHFR activity) results in their demise. Over 24 weeks, this results in a detectable reduction in circulating T cell numbers in PWH. We hypothesize that by targeting the most activated, proliferative, and potentially inflammatory cells, this effect eventually manifests as a clinical anti-inflammatory benefit.

Folinic acid administration completely reversed the MTX-mediated inhibition of proliferation *in vitro*, suggesting that MTX acted *via* inhibition of DHFR. This may be different than the effects of MTX in RA, in which MTX may act *via* other mechanisms instead of or in addition to DHFR inhibition (18). Indeed, it has been postulated that the anti-inflammatory effects of LDMTX in RA are separate from its DHFR activity, but rather are due to other mechanisms including adenosine accumulation, impaired T cell activation, and even inhibition of IL-1β/IL-1β receptor interactions (6, 24, 25). The lack of an effect of LDMTX on IL-6 or CRP levels in either A5314 or CIRT is evidence against an IL-1β-mediated mechanism (12, 14). Our *in vivo* data showing reduced CD38/HLA-DR co-expression on CD8 T cells does fit with impaired T cell activation, but *in vitro* we found minimal direct effect of MTX on stimulation-induced activation. Rather, it seems that the continuous death of activated cells driven to enter cell cycle results in the apparent decrease of activated cells overall. Regarding adenosine accumulation, while we confirmed that AMP inhibited TCR-induced proliferation *via* CD73 activity, which converts AMP to adenosine, folinic acid could not reverse adenosine-mediated inhibition of proliferation, nor could CD73 inhibition restore proliferation following MTX exposure, suggesting that these are separate inhibitory pathways.

MTX has also been proposed to act as a JAK/STAT inhibitor, including by inhibiting the phosphorylation and activation of STAT5 (26). We and others have shown that inhibiting STAT5 phosphorylation in CD8 T cells impairs their proliferation and reduces mTOR-mediated phosphorylation of S6 kinase and ribosomal S6 protein (20, 21). Here we found that MTX did not affect mitochondrial activity or phosphorylation of either STAT5 or ribosomal S6 protein after stimulation with IL-15, a robust activator of STAT5. The mTOR signaling network can sense changes in cellular levels of purine nucleotides, and a lack of purines inhibits mTOR complex 1 (mTORC1) signaling, measured in part by reduced phospho-S6 (pS6) accumulation (27). Yet again, we saw no effect here on pS6 levels after MTX exposure following either IL-15 stimulation or TCR engagement, suggesting that the inhibitory effects of MTX in our system are unlikely to be due to reduced availability of purine nucleotides.

Taken together, these observations support the conclusion that the anti-inflammatory effects of LDMTX *in vivo* are mediated indirectly by interfering with the proliferation of activated T cells *via* DHFR inhibition, and not by inducing the release of extracellular ATP or AMP or by inhibiting inflammatory cytokine production on a per-cell level. Importantly, because participants in A5314 also received 1mg folic acid daily throughout the study, our data also suggest that this dose of folic acid or its timing is insufficient to rescue the anti-proliferative effect of LDMTX *in vivo* in persons with HIV. Neither folic acid nor folinic acid supplementation reverses the anti-inflammatory effects of LDMTX in people with RA, except when given very proximate to the MTX dose (28–35). Whether our findings are specific to PWH who maintain robust levels of cycling T cells or is more generalizable is an open question, as is a better understanding of the mechanisms that drive T cell cycling in PWH (36, 37). While the effects of MTX administration on T cell cycling have not been reported for the CIRT trial – which did not include PWH – leukopenia was observed more often among patients in the LDMTX arm (12). Our *in vitro* data show that the 50% inhibitory concentrations (IC50s) of MTX are largely similar regardless of the T cell type, the donor HIV serostatus, and whether the proliferation was induced by cytokines or TCR engagement.

The reduction of cycling cells that lack Bcl-2 is an intriguing observation that may have relevance for understanding the activation of T cells during ART-treated HIV infection in general, and for the maintenance of the HIV reservoir specifically. For instance, recent work has suggested that Bcl-2 provides a survival signal for CD4 T cells that contain integrated HIV provirus, protecting them from cytolytic CD8 T cell targeting (38, 39). As the HIV reservoir is maintained by at least in part by proliferation of latently-infected CD4 T cells (40, 41), LDMTX administration in PWH may not ultimately result in reservoir reduction due to preferential survival of latently-infected CD4 T cells expressing Bcl-2.

There were some other potential therapeutic effects of LDMTX in PWH. VCAM levels fell modestly and LDMTX treatment may have improved arterial inflammation and structure as measured by FDG PET/CT imaging and brachial artery GLDS-CON, respectively, and early evidence suggests these changes may be linked to the effects of LDMTX on T cells (15–17). Indeed, a mechanistic role for CD8 T cells in the development of ASCVD is suggested by our recent data demonstrating that CD8 T cells localize to sites of endothelial dysfunction in the aortas of simian immunodeficiency virus (SIV) and simian-HIV (SHIV)-infected rhesus macaques and in atherosclerotic vascular tissues of HIV-uninfected donors (42, 43). These cells could contribute to local inflammation by their production and release of cytokines such as IFNγ, TNF, and macrophage inflammatory protein-1β, and they may promote coagulopathy *via* TNF-mediated induction of tissue factor on myeloid cells (20, 43, 44). If activation of these vascular CD8 T cells leads to their proliferation, then impairment of their viability by LDMTX treatment could ultimately reduce inflammation and cardiovascular events. However, the failure of the CIRT study to reduce CVD events (12) raises questions about this mechanism and how much these pro-inflammatory vascular CD8 T cells may be driven to proliferate *in situ*, at least in those without HIV infection.

There are some notable limitations of our study. First, while we did characterize samples from the A5314 clinical trial of LDMTX by flow cytometry *ex vivo*, all *in vitro* mechanistic experiments were performed using cells from PWH or HIV-uninfected controls who were not taking MTX, so it is unclear if similar results would be observed if the cells were exposed to MTX *in vivo*. Second, we do not have data on the effects of MTX on cells from participants with RA or psoriasis, in whom the mechanisms of MTX action may differ. Third, we did not measure the levels of adenosine receptors on cells from A5314 trail participants, nor did we directly inhibit adenosine receptor activity in our *in vitro* assays. Along these same lines, we did not formally address the effects of MTX on 5-aminoimidazole-4-carboxamide (AICA) or AICA ribonucleotide (AICAR) transformylase (ATIC), and it is possible that inhibition of ATIC by MTX could lead to increased levels of adenosine regardless of CD73 activity (45). However, in our assays, folinic acid fully reversed the anti-proliferative effects of MTX without reversing the CD73-depenedent anti-proliferative effects of AMP, suggesting that if MTX was also acting *via* ATIC inhibition and adenosine accumulation, we would have observed only a partial restoration of proliferative capacity with folinic acid. Finally, all participants in A5314 received supplementation with folic acid; whether supplementation with folinic acid, which bypasses DHFR, would have attenuated the *in vivo* effects of LDMTX in PWH is unknown.

In conclusion, our detailed analysis of samples from a randomized, placebo-controlled clinical trial of LDMTX in PWH coupled with *in vitro* assays using cells from PWH and HIV-infected controls demonstrates that the anti-inflammatory efficacy of MTX is likely indirect *via* inhibition the proliferation and survival of activated pro-inflammatory CD4 and CD8 T cells. The failure of folic acid administration (1mg daily) to prevent this effect *in vivo* is an interesting observation. Whether higher doses of folic or folinic acid would prevent this effect without attenuating the clinical activity of MTX and whether similar cytopenic effects would be seen in persons with RA or psoriasis treated with LDMTX are important questions.

## Data Availability Statement

The original contributions presented in the study are included in the article/**Supplementary Material**. Further inquiries can be directed to the corresponding authors.

## Ethics Statement

The studies involving human participants were reviewed and approved by Institutional Review Board of University Hospitals, Cleveland Medical Center (IRB #01-98-55). The patients/participants provided their written informed consent to participate in this study.

## Author Contributions

MF conceived the study, performed experiments, analyzed data, and wrote the manuscript. BC, DM, CM, and AR performed experiments and analyzed data. EY analyzed data and was a statistician for the A5314 clinical trial. BR contributed to study design and facilitated sample acquisition. JS, SD, JC, and PH designed, conducted, and secured funding for the A5314 clinical trial. DA and LC contributed to study design. HR analyzed data and was a statistician for the A5314 clinical trial. ML conceived the study, secured funding, supervised immunologic monitoring of the A5314 clinical trial, and wrote the manuscript. All authors critically reviewed the manuscript. All authors contributed to the article and approved the submitted version.

## Funding

Research reported in this publication was supported by the National Institute of Allergy and Infectious Diseases (NIAID) of the National Institutes of Health (NIH) under award numbers UM1 AI068634, UM1 AI068636 and UM1 AI106701, and by the National Heart, Lung, and Blood Institute (NHLBI) of the NIH under award number R01 HL117713.

## Author Disclaimer

The content is solely the responsibility of the authors and does not necessarily represent the official views of the NIH.

## Conflict of Interest

JS has received personal fees from Lilly for being on a data and safety monitoring board and a core laboratory grant from Novartis. JC has received grants from Theratechnologies. PH has received honoraria from Gilead and Merck. LC has consulted for Genentech-Roche. ML has received competitive funding from Gilead Sciences and consulted for Lilly.

The remaining authors declare that the research was conducted in the absence of any commercial or financial relationships that could be construed as a potential conflict of interest.

## Publisher’s Note

All claims expressed in this article are solely those of the authors and do not necessarily represent those of their affiliated organizations, or those of the publisher, the editors and the reviewers. Any product that may be evaluated in this article, or claim that may be made by its manufacturer, is not guaranteed or endorsed by the publisher.
