# Supplementary material for: Methotrexate Inhibits T Cell Proliferation but Not Inflammatory Cytokine Expression to Modulate Immunity in People Living With HIV
Source: Front Immunol. 2022 Jul 29;13:924718. doi: 10.3389/fimmu.2022.924718 (PMC9374564; doi:10.3389/fimmu.2022.924718)
Supplement: Supplementary file 1 [file DataSheet_1.pdf]

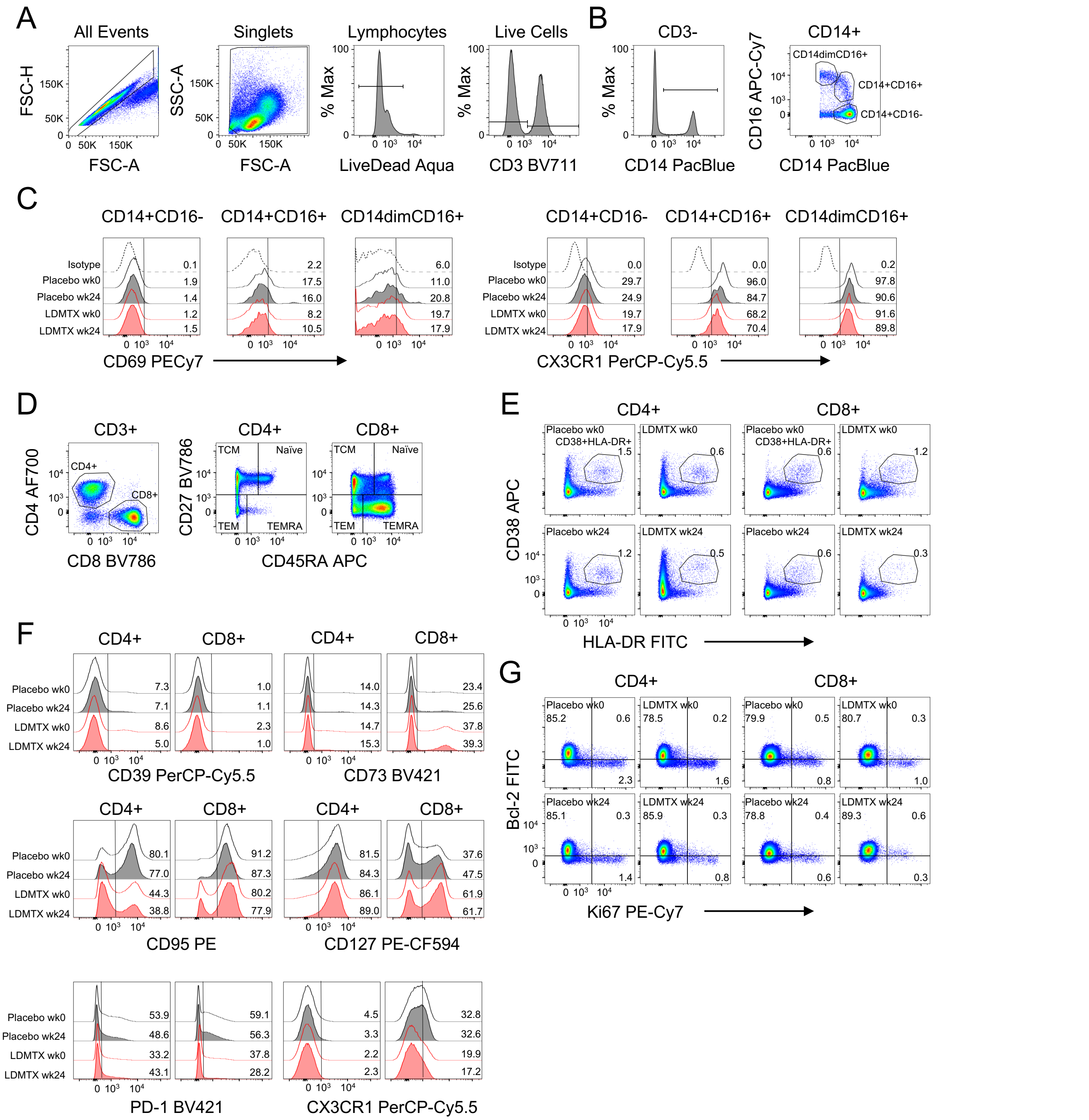

Supplementary Fig. 1: Representative flow cytometry. (A) Pseudocolor plots and histograms showing overall gating strategy. Sample is from a placebo-treated individual at week 0 (baseline). **(B)** Monocyte gating strategy. **(C)** Expression of CD69 and CX3CR1 on monocyte subsets. Isotype control staining is shown for comparison. **(D)** T cell and maturation subsets gating strategy. **(E)** Expression of CD38 and HLA-DR on CD4 and CD8 T cells. **(F)** Expression of CD39, CD73, CD95, CD127, PD-1, and CX3CR1 on CD4 and CD8 T cells. **(G)** Expression of Bcl-2 and Ki67 on CD4 and CD8 T cells. Gates were set on isotype controls or on populations known to be negative.

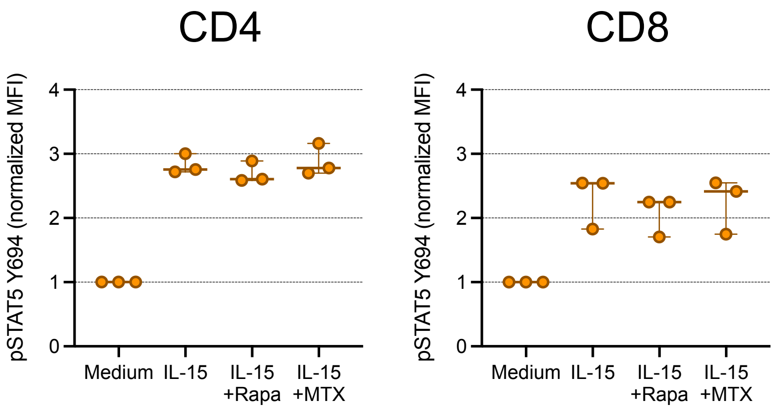

Supplementary Figure 2: MTX does not inhibit IL-15-induced STAT5 phosphorylation.

Cryopreserved PBMCs from HIV-uninfected controls (n=3) were stimulated for 45 minutes with IL-15 (24ng ml^{-1}) in the presence or absence of MTX (100nM) or rapamycin (rapa; 250ng ml^{-1}). Normalized MFI of STAT5 phosphorylated at Y694 is shown among CD4 (left) and CD8 (right) T cells.

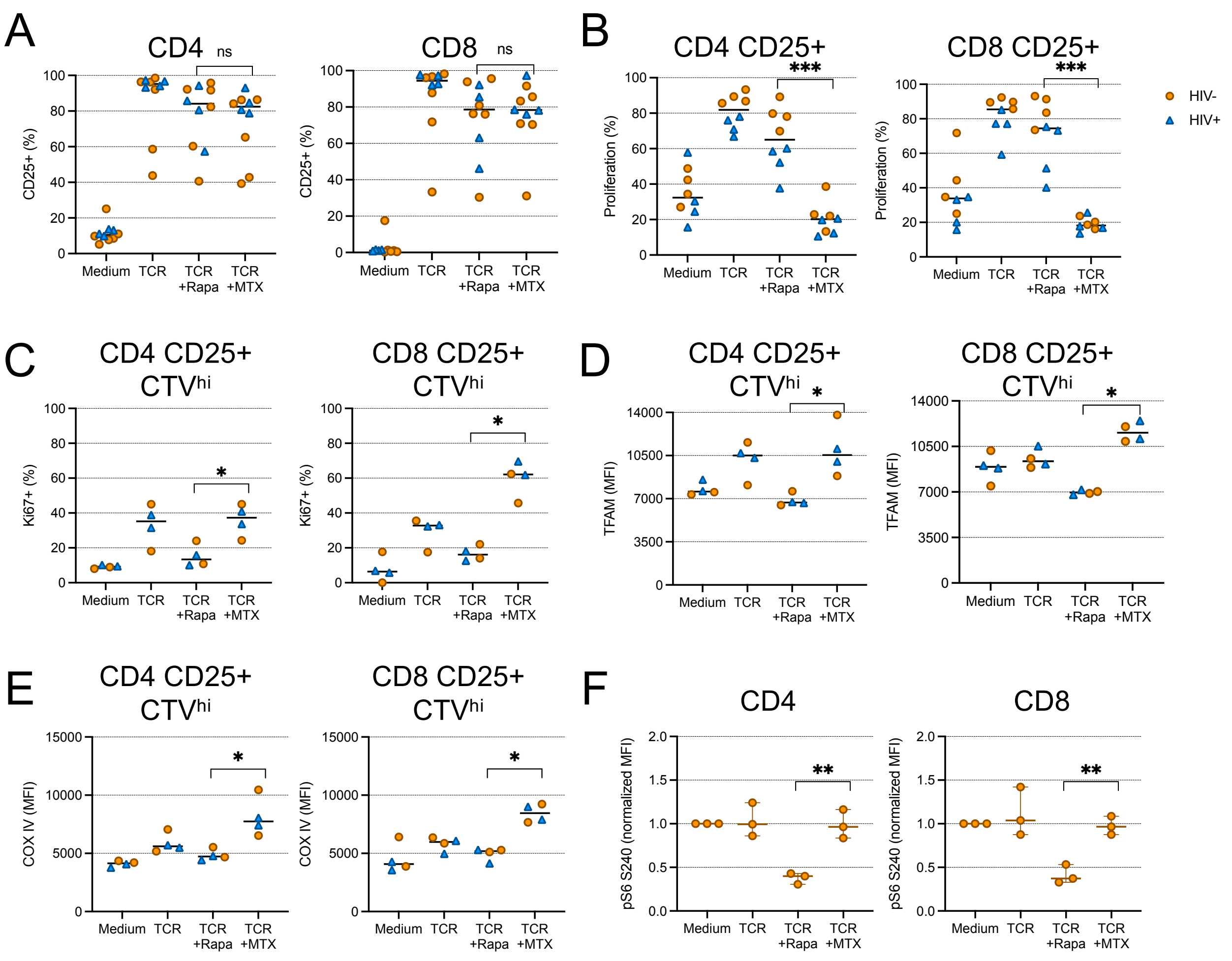

Supplementary Fig. 3: MTX inhibits TCR-induced proliferation at a step after cell cycle entry and mTOR activity.

Cryopreserved PBMCs from PWH (blue triangles, n=2-4) or HIV-uninfected controls (gold circles, n=2-6) were labeled with CellTraceViolet (CTV) then stimulated for 4 days **(A-E)** or 45 minutes **(F)** with anti-CD3/anti-CD28 (TCR) in the presence or absence of MTX (100nM) or rapamycin (rapa; 250ng ml^{-1}). At the end of the culture, cells were harvested and analyzed by flow cytometry. **(A)** The proportion of CD4 (left) and CD8 (right) T cells expressing CD25. **(B)** The proportion of CD4 (left) and CD8 (right) CD25+ cells that diluted CTV dye. **(C)** The proportion of CD4 (left) and CD8 (right) cells that did not dilute CTV dye (CTV^{hi}) that expressed Ki67. **(D)** The mean fluorescence intensity (MFI) of mitochondrial TFAM among CD4 (left) and CD8 (right) CD25+ CTV^{hi} cells. **(E)** The MFI of mitochondrial COX IV among CD4 (left) and CD8 (right) CD25+ CTV^{hi} cells. **(F)** Normalized MFI of ribosomal S6 protein phosphorylated at S240 among CD4 (left) and CD8 (right) T cells. Statistical significance determined between TCR+rapa and TCR+MTX groups; Mann-Whitney test. ns, not significant; *P<0.05; **P<0.01; ***P<0.001.

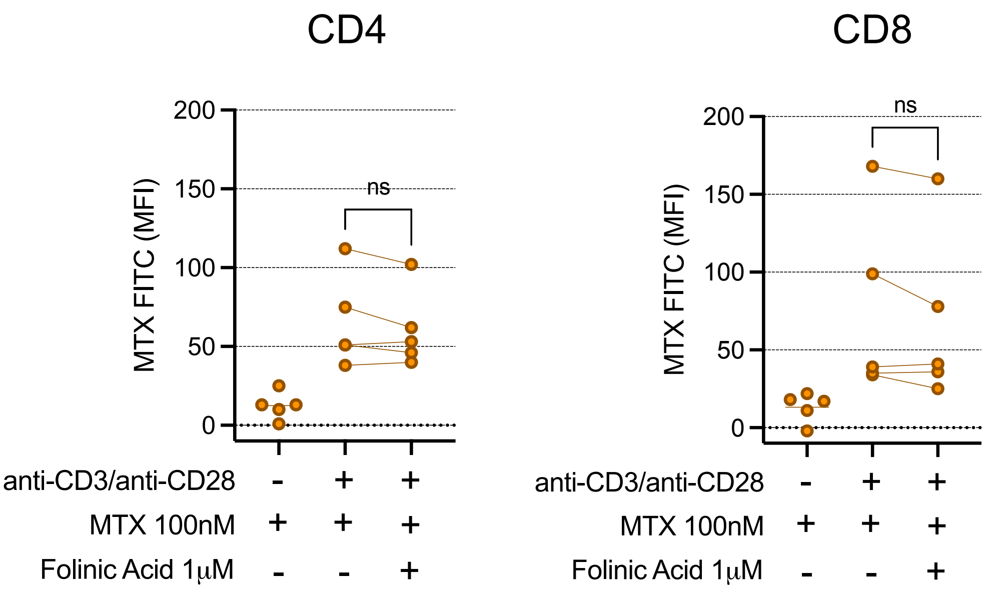

Supplementary Figure 4: Folinic acid does not inhibit MTX uptake by activated T cells.
Cryopreserved PBMCs from HIV-uninfected controls (n=5) were stimulated overnight with anti-CD3/anti-CD28 or medium. The following morning, folinic acid (1μM) and/or fluorescein-labeled MTX (MTX FITC, 100nM) was added. After 45 minutes, the cultures were harvested and CD4 (left) and CD8 (right) T cells were analyzed by flow cytometry. The mean fluorescence intensity (MFI) of MTX FITC is shown. Statistical significance determined using nonparametric Wilcoxon matched pairs signed rank test. ns, not significant.

Supplementary Table 1. Median changes from baseline for circulating monocyte subsets and activation marker expression in A5314.

	Treatment				
	LDMTX (N=57)	Placebo (N=69)	LDMTX (N=56)	Placebo (N=68)	P-value
	Week 0	Week 0	Δ to Week 24	Δ to Week 24	Week 24
CD14dim CD16+, %[a] (Q1, Q3)	3.98 (2.21, 6.23)	3.51 (2.33, 5.84)	-0.10 (-1.20, 1.76)	-0.09 (-1.34, 1.19)	0.65
…. CD69+, % (Q1, Q3)	2.70 (1.70, 5.30)	2.50 (1.50, 4.90)	0.05 (-1.70, 1.20)	0.30 (-1.00, 2.30)	0.33
…. CX3CR1+, % (Q1, Q3)	93.5 (87.4, 97.2)	93.8 (85.7, 96.3)	0.02 (-3.75, 1.71)	0.34 (-2.13, 3.66)	0.31
CD14+CD16+, % (Q1, Q3)	3.81 (2.48, 5.52)	3.79 (2.59, 5.22)	-0.06 (-1.19, 1.46)	-0.11 (-1.40, 0.87)	0.57
…. CD69+, % (Q1, Q3)	6.30 (2.90, 12.6)	6.00 (2.60, 13.9)	1.10 (-4.35, 4.65)	1.45 (-2.45, 6.70)	0.41
…. CX3CR1+, % (Q1, Q3)	77.1 (63.4, 84.3)	74.6 (65.8, 86.0)	0.22 (-6.87, 7.85)	0.40 (-6.01, 8.65)	0.64
CD14+CD16-, % (Q1, Q3)	91.8 (88.6, 94.3)	92.7 (87.1, 95.2)	0.30 (-2.70, 2.50)	0.18 (-2.16, 2.73)	0.88
…. CD69+, % (Q1, Q3)	14.3 (9.00, 23.4)	14.7 (8.70, 27.7)	0.20 (-4.90, 6.55)	2.25 (-4.35, 10.3)	0.30
…. CX3CR1+, % (Q1, Q3)	45.5 (31.9, 56.0)	46.6 (34.4, 55.6)	-0.54 (-6.87, 7.93)	0.07 (-5.50, 7.48)	0.73

Supplementary Table 2. Changes from baseline in soluble inflammatory biomarkers in A5314 participants.

	Treatment				
	LDMTX (N=55)	Placebo (N=69)	LDMTX (N=55)	Placebo (N=69)	P-value
	Week 0	Week 0	Δ to Week 24	Δ to Week 24	Week 24
CX3CL1, pg ml^{-1}, median (Q1, Q3)	937 (816, 1072)	946 (758, 1070)	1.1% (-8.6, 14.0)	-2.1% (-11.4, 9.9)	0.33
IL-18, pg ml^{-1}, median (Q1, Q3)	204 (159, 260)	229 (183, 292)	2.3% (-9.4, 15.2)	0.4% (-11.8, 12.8)	0.63
β2 microglobulin, μg ml^{-1}, median (Q1, Q3)	2.20 (1.82, 2.74)	2.34 (1.98, 2.55)	2.4% (-9.1, 10.6)	-0.1% (-7.1, 9.1)	0.66
IL-7, pg ml^{-1}, median (Q1, Q3)	3.46 (166, 5.95)	3.99 (1.73, 6.26)	1.5% (-26.6, 51.3)	4.0% (-25.7, 59.3)	0.78

Supplementary Table 3. IC50 of MTX inhibition of proliferation.

	HIV- (N=8-11)	HIV+ (N=6)	P-value
	IC50 (95% CI)	IC50 (95% CI)	
CD4 T cells Day 4			
TCR	40.63 (34.54 - 48.16)	34.23 (27.82 - 42.70)	0.37
IL-2	106.2 (60.35 - 1311)	60.96 (26.45 - undefined)	0.19
IL-7	105.9 (68.56 - 338.2)	109.0 (62.64 - 761.5)	0.34
IL-15	70.14 (42.08 - 259.9)	60.49 (38.81 - 145.8)	0.59
CD8 T cells Day 4			
TCR	43.45 (34.77 - 55.19)	36.75 (30.34 - 45.01)	0.54
IL-2	1307 (129.7 – undefined)	64.96 (27.87 – undefined)	**0.0131**
IL-7	136.2 (73.03 – undefined)	197.4 (101.9 - 5091)	0.73
IL-15	55.05 (36.15 - 102.2)	23.79 (15.81 - 34.09)	**0.0156**
